# Heterogeneity and Glycan Masking of Cell Wall Microstructures in the Stems of *Miscanthus* x *giganteus*, and Its Parents *M. sinensis* and *M*. *sacchariflorus*

**DOI:** 10.1371/journal.pone.0082114

**Published:** 2013-11-29

**Authors:** Jie Xue, Maurice Bosch, J. Paul Knox

**Affiliations:** 1 Centre for Plant Sciences, Faculty of Biological Sciences, University of Leeds, Leeds, United Kingdom; 2 Institute of Biological, Environmental and Rural Sciences, Aberystwyth University, Plas Gogerddan, Aberystwyth, United Kingdom; Nanjing Agricultural University, China

## Abstract

Plant cell walls, being repositories of fixed carbon, are important sources of biomass and renewable energy. *Miscanthus* species are fast growing grasses with a high biomass yield and they have been identified as potential bioenergy crops. *Miscanthus* x *giganteus* is the sterile hybrid between *M. sinensis* and *M. sacchariflorus*, with a faster and taller growth than its parents. In this study, the occurrence of cell wall polysaccharides in stems of *Miscanthus* species has been determined using fluorescence imaging with sets of cell wall directed monoclonal antibodies. Heteroxylan and mixed linkage-glucan (MLG) epitopes are abundant in stem cell walls of *Miscanthus* species, but their distributions are different in relation to the interfascicular parenchyma and these epitopes also display different developmental dynamics. Detection of pectic homogalacturonan (HG) epitopes was often restricted to intercellular spaces of parenchyma regions and, notably, the high methyl ester LM20 HG epitope was specifically abundant in the pith parenchyma cell walls of M. x *giganteus*. Some cell wall probes cannot access their target glycan epitopes because of masking by other polysaccharides. In the case of *Miscanthus* stems, masking of xyloglucan by heteroxylan and masking of pectic galactan by heteroxylan and MLG was detected in certain cell wall regions. Knowledge of tissue level heterogeneity of polysaccharide distributions and molecular architectures in *Miscanthus* cell wall structures will be important for both understanding growth mechanisms and also for the development of potential strategies for the efficient deconstruction of *Miscanthus* biomass.

## Introduction

Plant cell walls are important cellular components that perform a number of critical functions in relation to cell morphology, cell differentiation and tissue and organ enlargement [1,2]. Cell walls, being the major repository for photosynthetically-fixed carbon, are one of the major resources of renewable biomass on Earth. Certain C4 grasses such as switchgrass, sorghum and *Miscanthus* species, with fast growth and high capacity for biomass accumulation, are potential targets to develop as bioenergy crops [3].

 Cell walls are complex fibrous composites constructed from a range of glycans and the structurally complex and tightly compacted nature of cell walls results in them not being amenable to facile enzymatic deconstruction to release sugars. This cell wall recalcitrance is a major hurdle in the optimization of cell wall biomass and therefore understanding cell wall microstructures and cell wall heterogeneity is an important step in their exploitation [4-6]. However, surprisingly little is known of cell and tissue cell wall heterogeneity in the vegetative organs of grass species. Cellulose, consisting of hydrogen-bonded chains of β-1,4-D-linked-glucosyl residues, forms crystalline microfibrils that provide high mechanical strength and rigidity to plants [7] and is distributed at broadly similar levels in cell walls of all land plants. Within the commelinid group of monocotyledons, and specifically the Poaceae family of grasses, taxonomically restricted configurations of cell wall polysaccharides are known in which major non-cellulosic polymers are heteroxylan (glucuronoarabinoxylan, GAX) and mixed-linkage glucan (MLG) with lower levels of xyloglucan [6,8-11]. An additional feature is the presence of phenolics such as ferulic acid attached to heteroxylan polymers that can function in cell wall polymer cross-linking and this can contribute to cell wall recalcitrance [12,13]. The galacturonic acid-rich pectic polysaccharides are complex supramolecular components of cell wall matrices and include the homogalacturonan (HG), rhamnogalacturonan-I (RG-I), rhamnogalacturonan-II (RG-II) and xylogalacturonan (XGA) domains [14]. Pectic polymers are generally proposed to be present at lower levels in grass cell walls (~10% of polymers) relative to the cell walls of dicotyledons and non-commelinid monocotyledon species (~30% of polymers) [8,15]. 

*Miscanthus* species are grasses which are native to tropical and subtropical regions of southern Asia and Africa and some *Miscanthus* species have been used as bioenergy crops in Europe since the early 1980s. *Miscanthus* x *giganteus* has rapid growth, low mineral content, and high biomass yield [16] and is a major target for study and analysis *M*. x *giganteus* is the sterile hybrid between *M. sinensis*, an ornamental grass, and *M. sacchariflorus* [17]. M. x *giganteus* grows faster and taller than *M. sinensis* and *M. sacchariflorus* and can be clonally propagated from rhizome cuttings to generate mature stands that provide yields which can be maintained for 20 or more years of production [18]. *Miscanthus* biomass can also be used in the paper industry, pharmaceutical industry and for water and soil conservation [19]. Some aspects of the anatomy and chemistry of stems of various *Miscanthus* genotypes have been reported [20] and some cell wall composition data are known which indicate that glucose, xylose and arabinose are the most abundant neutral monosaccharides and that heteroxylans/GAXs comprise ~35% and MLG ~2% of cell wall materials of mature plants [17,21-23]. However, the distributions of cell wall polysaccharides within cell walls of *Miscanthus* species in the context of cells, tissues, cell wall architectures and cell functions during growth have not been reported. 

 Molecular probes (such as monoclonal antibodies), targeted to cell wall glycans, are specific and sensitive detection tools that can be used in conjunction with fluorescence imaging to determine cell wall microstructures and thus any heterogeneities between cell walls or cell wall regions [1,24-27]. Recent work using immunohistochemical approaches to study cell wall structures *in situ* has indicated that in some instances the detection of a particular polysaccharide epitope can be blocked or masked by the presence of other polysaccharides [28-30]. To date, this phenomenon, which indicates a fundamental aspect of cell wall microstructure and also provides insights in the capacity of proteins to access target ligands or substrate polysaccharides within cell walls, has only been reported for cell walls of dicotyledons. Here, we use sets of cell wall directed probes and enzymes to study the occurrence and configurations of cell wall polysaccharides in the context of the stem anatomies of M. x *giganteus*, *M. sacchariflorus* and *M. sinensis*. 

## Materials and Methods

### Plant material and its preparation for immunomicroscopy

The *Miscanthus* species used were M. x *giganteus* clone Illinois, *M. sacchariflorus* (Sac-177), and *M. sinensis* (Sin-183). Plants were grown in 5 L pots containing soil and Osmocote Exact standard 5-6M controlled release fertilizer (Scotts, Australia), with 16 h days (600-750 µmol/m^2^/s) at 20°C. Most analyses focused on stem material obtained from the middle of the second internode, counting from the base, after 50 days of growth. In some cases, material was also analysed from the top and base of the second internode and also from the third, fourth and fifth internodes counting from the base. In all cases, 2-cm regions of the internodes were excised, fixed in PEM buffer (50 mM piperazine-N,N'-bis[2-ethane-sulfonic acid] (PIPES), 5 mM methylene glycol bis(β-aminoethylether)-*N,N,N*',N'-tetraacetic acid (EGTA), 5 mM MgSO_4_ (pH 6.9)) containing 4% paraformaldehyde and vacuum infiltrated using a vacuum pump for 60 min. All steps were carried out at room temperature. The fixed excised regions were dehydrated with a graded ethanol series (30%, 50%, 70%, 90%, and 100%) for 40 min each at 4°C. For the preparation of Steedman’s wax, 900 g of polyethylene glycol 400 distearate (Sigma 30, 541-3) and 100 g 1-hexadecanol (Sigma C7882) were incubated at 65°C until melted. The wax was thoroughly mixed and poured into an aluminium foil lined tray and allowed to cool. Samples were incubated in 1:1 Steedman’s wax and 100% ethanol at 37°C overnight, followed by two changes of 100% wax for 1 h at 37°C. The samples were placed into moulds, and molten wax poured over until a convex surface was visible. Moulds were left to set overnight at room temperature. Using a Microm HM-325 microtome, transverse sections were cut to a thickness of 12 µm and placed onto glass slides coated with polysine (VWR international, Leuven, Belgium). Slides were dewaxed in a graded ethanol series (3x 97%, 90%, 50%, 2x water) and allowed to dry before immunolabelling procedures.

### Molecular probes for cell wall analyses

The monoclonal antibody probes used in this study were the rat monoclonal antibodies: LM10, LM11, that bind to epitopes of heteroxylan [25]; LM12 directed to ferulate residues and in *Miscanthus* species would bind to feruloylated xylan [31]; LM15 to the XXXG structural motif of xyloglucan [28]; LM21 to heteromannan [29]; LM19 to low/no ester pectic HG and LM20 to high ester pectic HG [26]; LM5 to pectic (1→4)-β-galactan [32]; LM6 to pectic (1→5)-α-arabinan [33] and mouse monoclonal antibody BG1 to MLG [24]. 

### Immunocytochemistry including enzymatic pretreatments

Transverse sections of *Miscanthus* stem internodes were incubated for 30 min with 5% (w/v) milk protein/phosphate-buffered saline (MP/PBS) to prevent non-specific binding, and then washed for 5 min with PBS. Primary rat monoclonal antibodies at 5-fold dilutions of hybridoma cell culture supernatants in MP/PBS (5 µg/ml for the mouse antibody BG1) were incubated on sections for 90 min at RT. Sections were then washed three times with PBS for 5 min. The secondary antibodies (anti-rat IgG-FITC (Sigma-Aldrich, UK) at a 100-fold dilution for the rat primary antibodies and anti-mouse IgG-FITC (Sigma-Aldrich, UK) at a 50-fold dilution for the BG1 MLG primary antibody) were added in 5% MP/PBS and incubated for 90 min in the dark. Sections were washed with PBS for three times for 5 min. After immunolabelling some sections were incubated with Calcofluor White (CW, Fluorescent Brightner 28, Sigma-Aldrich, UK, 0.2 mg/mL in PBS) for 5 min in the dark. To diminish sample auto-fluorescence some sections were incubated with 0.1% Toluidine Blue O (pH 5.5, 0.2 M sodium phosphate buffer) for 5 min in place of CW. Following CW or Toluidine Blue O labelling, sections were washed twice with PBS each for 5 min, then mounted in anti-fade reagent Citifluor AF1 (Agar Scientific, UK). After mounting slides were stored at 4°C in darkness until use. Sections were observed with a fluorescence microscope (Olympus BX61) and images were captured with a Hamamatsu ORCA285 camera (Hamamatsu City, Japan) using PerkinElmer Volocity software (PerKinElmer, UK). 

 In some cases, stem sections were pre-treated, prior to immunolabelling, with enzymes to remove specific cell wall polysaccharides. Removal of pectic HG and heteroxylan was carried out as described [34] using pectate lyase (*Aspergillus* sp. Megazyme International, Bray, Ireland) in 50 mM 3-(cylohexylamino)-1-propanesulfonic acid (CAPS), 2 mM CaCl_2_ buffer, pH 10 at 25 μg/ml 2 h at room temperature and xylanase (*Cellvibrio japonicus*, a gift from Prof Harry Gilbert, Newcastle University) at 20 μg/ml in 25 mM Na-acetate buffer, pH 5.5 overnight at RT. Lichenase (*Bacillus subtilis* Megazyme International, Bray, Ireland) was used at 20 μg/ml in 100 mM sodium acetate buffer pH 5.0, at RT. Xyloglucanase (*Paenibacillus* sp. Megazyme International, Bray, Ireland) was used at 20 μg/ml in PBS overnight, at RT). Control sections not treated with enzymes were incubated for an equivalent time with the corresponding buffers alone. 

Micrographs shown in figures are representative of at least 9 sections for each point of analysis (derived from the analysis of at least three sections across the internode obtained from each of at least three separate plants). Negative control, no antibody, micrographs are shown in the supporting information. Micrographs of unmasked epitopes are representative of at least 10 separate deconstruction experiments. All raw image data are available upon request from the corresponding author. 

## Results

### Heterogeneities in detection of non-cellulosic polysaccharides indicates distinct stem parenchyma cell wall microstructures in M. *sacchariflorus*

**Figure 1:**
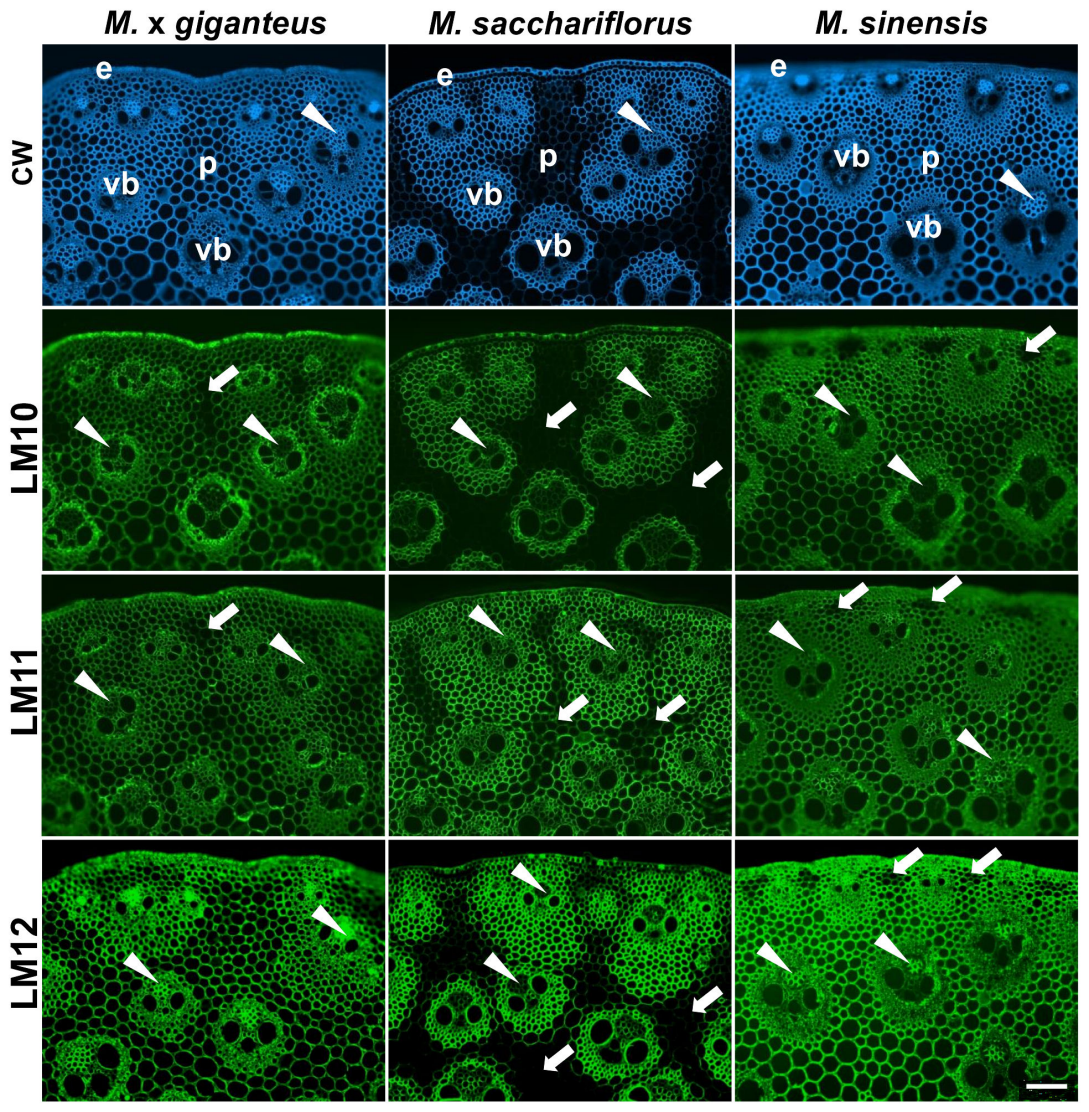
Fluorescence imaging of cell walls in equivalent transverse sections of the second internode of stems of *M*. ***xgiganteus*, *M***. ***sacchariflorus* and *M. sinensis* at 50 days growth.** Images generated with Calcofluor White (CW, blue) and indirect immunofluorescence (green) with monoclonal antibodies to epitopes of heteroxylan LM10, LM11 and LM12. e = epidermis, p = parenchyma, vb = vascular bundle. Arrowheads indicate phloem. Arrows indicate regions of interfascicular parenchyma that have relatively lower levels of heteroxylan detection. Bar = 100 µm.

Calcoflour White (CW), which binds to cellulose and other β-glycans and fluoresces under UV excitation, is generally a highly effective stain to visualise all cell walls in sections of plant materials. The staining of equivalent transverse sections of the outer stem regions of the middle of the second internode from the base of a 50-day-old stem of M. x *giganteus*, *M. sacchariflorus* and *M. sinensis* are shown in Figure 1. At this growth stage the internodes are approximately 12 cm, 11 cm and 5 cm in length respectively. See Figure S1 in File S1 for details of materials analysed. In all three species an anatomy of scattered vascular bundles within parenchyma regions was apparent with the vascular bundles nearest to the epidermis being generally smaller in diameter to those in more internal regions. In all cases the vascular bundles consisted of a distal area of phloem cells (accounting for around a quarter of the vascular tissues) flanked by two large metaxylem vessels and a more central xylem cell in addition to surrounding sheaths of small fibre cells. The most striking distinction seen in the CW-stained sections was that in *M. sinensis* and *M*. x *giganteus*, CW-staining was equivalent in cell walls whereas in *M. sacchariflorus* the cell walls of the larger cells of the interfascicular parenchyma were not stained in the same way indicating some difference to the structure of these cell walls.

 The analysis of equivalent sections with three probes directed to structural features of heteroxylans, which are the major non-cellulosic polysaccharides of grass cell walls, indicated that these polymers were widely detected in *Miscanthus* stem cell walls (Figure 1). No antibody immunolabelling controls are shown in Figure S2 in File S1. The analysis also indicated that non-CW-staining cell walls in *M. sacchariflorus* had lower levels of detectable heteroxylan. This was particularly the case for the LM10 xylan epitope (unsubstituted xylan) and the LM12 feruloylated epitope both of which closely reflected the distribution of CW-staining (Figure 1). In the case of M. x *giganteus* some smaller regions of the interfascicular parenchyma were notable for reduced binding by the LM10 and LM11 xylan probes. In the case of *M. sinensis* such regions were most apparent as clusters of cells in sub-epidermal regions of parenchyma (Figure 1). 

**Figure 2:**
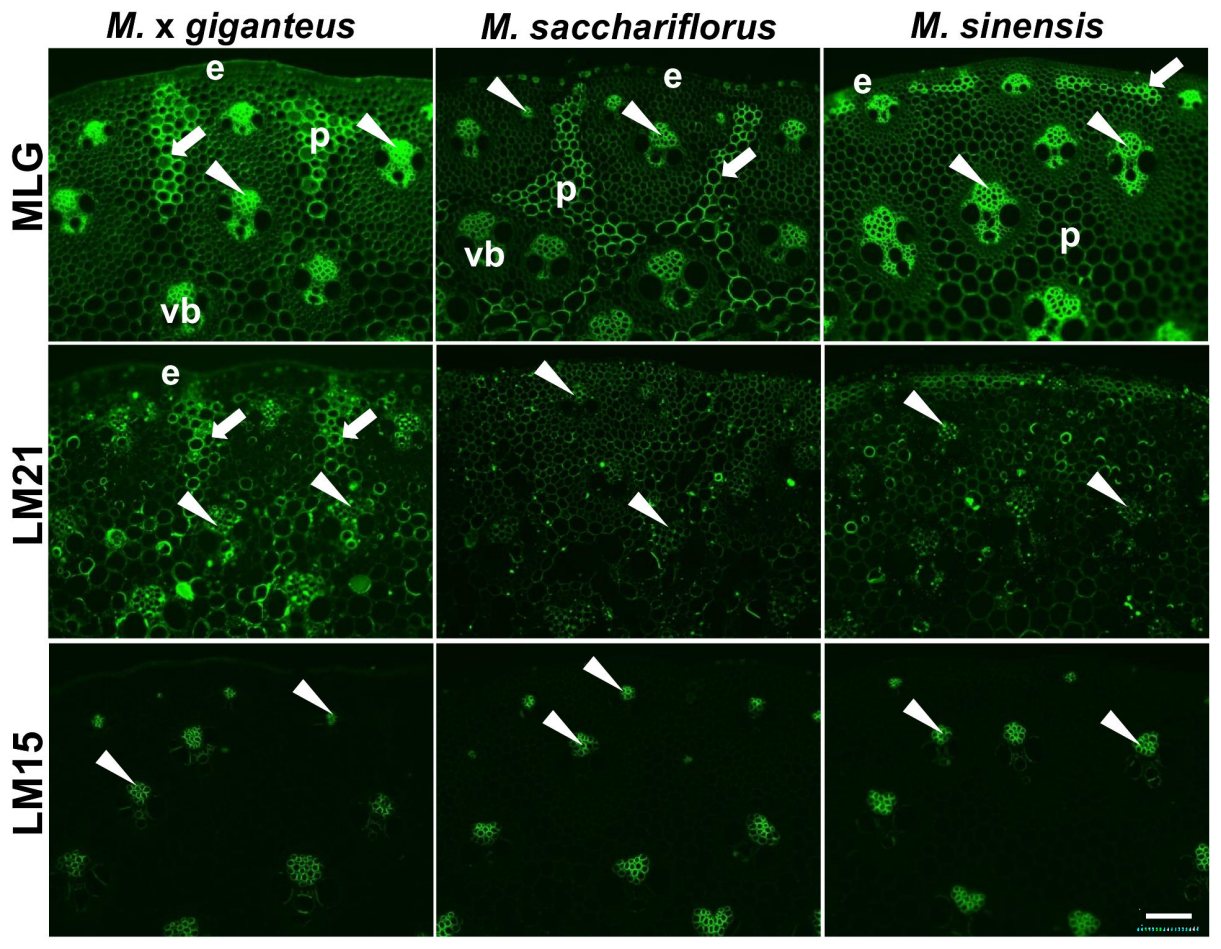
Fluorescence imaging of cell walls in equivalent transverse sections of the second internode of stems of *M*. ***xgiganteus*, *M***. ***sacchariflorus* and *M. sinensis* at 50 days growth.** Immunofluorescence images generated with monoclonal antibodies to MLG, heteromannan (LM21) and xyloglucan (LM15). e = epidermis, p = parenchyma, vb = vascular bundle. Arrowheads indicate phloem. Arrows indicate regions of interfascicular parenchyma that are labelled strongly by the probes. Bar = 100 µm.

 Analysis of equivalent sections with a monoclonal antibody directed to MLG also indicated some clear differences between the three species (Figure 2). In all three species the MLG epitope was detected with particular abundance in cell walls of phloem cells, the central metaxylem cells and in specific regions of the interfascicular parenchyma. Unlike the heteroxylan epitopes the MLG epitope was not abundantly detected in the fibre cells surrounding the vascular bundles. The specific patterns of abundant epitope detection in interfascicular parenchyma varied between the species but were consistent for each species. In M. x *giganteus*, the MLG epitope was strongly detected in radially extended groups of cells in the stem periphery. In *M. sinensis*, such groups of cells were smaller and were mostly sub-epidermal clusters of fewer than 10 cells. In *M. sacchariflorus* strong labelling was detected throughout the parenchyma regions. For all three species these parenchyma regions were equivalent to those with reduced staining by the heteroxylan probes. 

 The LM21 heteromannan epitope was only weakly detected in scattered cells in *M. sacchariflorus* and *M. sinensis* stem sections, reflecting the high MLG/low heteroxylan regions, was detected to some extent in phloem cell walls and more strongly to the MLG-rich parenchyma regions of M. x *giganteus*. The LM15 xyloglucan antibody bound specifically to phloem cell walls in all three species (Figure 2). In M. x *giganteus* and *M. sinensis* there was in addition some detection of the LM15 xyloglucan epitope in cell wall regions of the metaxylem cells (Figure 2). 

### Varied configurations of cell wall polymers in *Miscanthus* vascular cell walls

**Figure 3:**
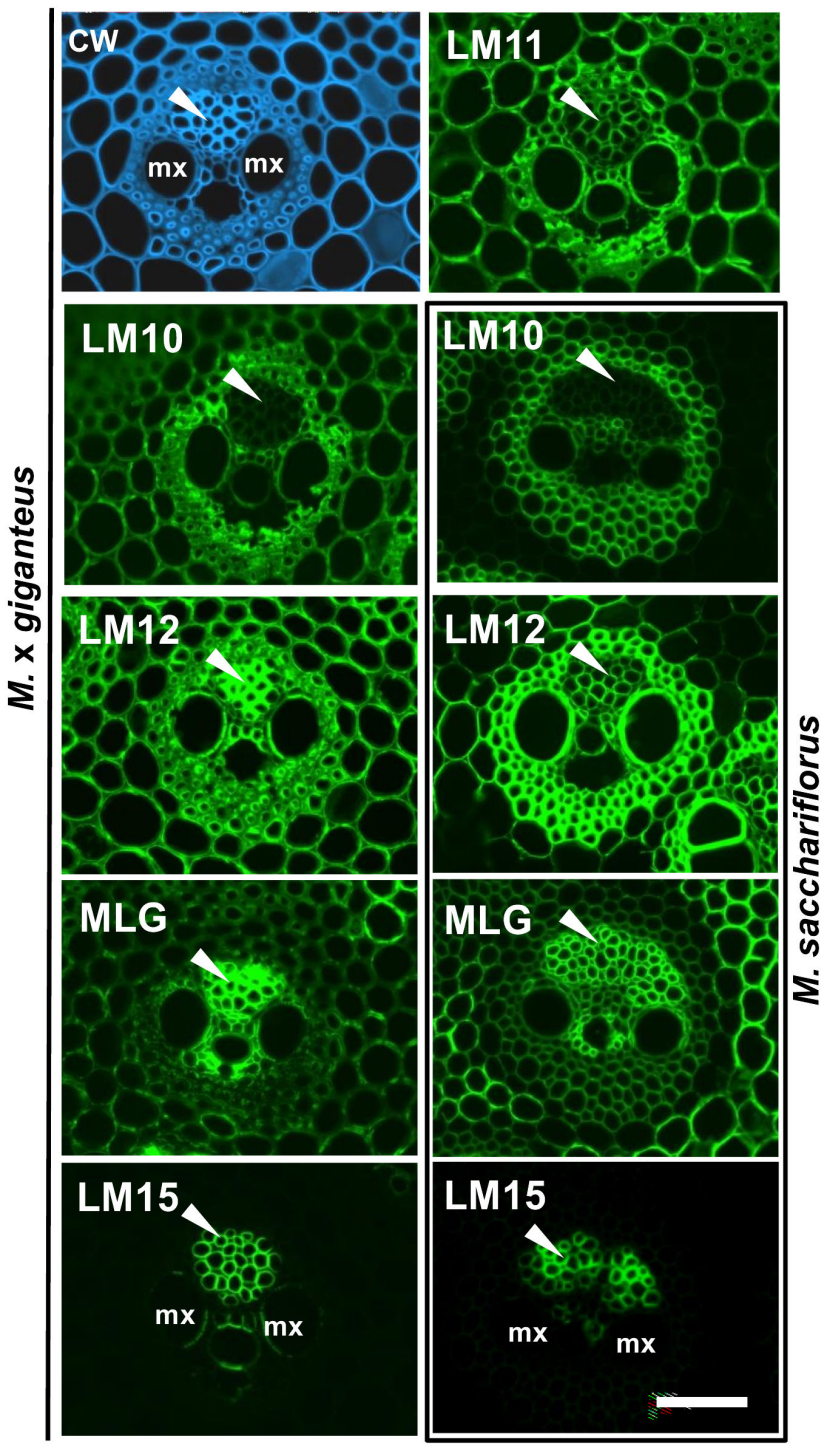
Fluorescence imaging of vascular bundles of the second internode of stems of *M*. ***xgiganteus* and *M***. ***sacchariflorus* at 50 days growth.** Immunofluorescence images generated with monoclonal antibodies to heteroxylan (LM10, LM11, LM12), MLG and xyloglucan (LM15). mx = metaxylem elements. Arrowheads indicate phloem. Bar = 50 µm.

The initial analyses indicated a range of cell wall heterogeneities in relation to the main non-cellulosic polysaccharides and several of these involved the cell types of the vascular bundles. Analysis of higher magnification micrographs (Figure 3) indicated that the phloem cell walls have abundant detectable LM11 xylan epitope but not the LM10 xylan epitope as shown for M. x *giganteus* in Figure 3. This was consistent for all three species (Figure 1). The LM12 ferulate epitope was notably highly detected in phloem cell walls of M. x *giganteus* and *M. sinensis* but less so in equivalent cells in *M. sacchariflorus* (Figures 1 and 3) whereas the MLG and LM15 xyloglucan epitopes were abundantly detected in phloem cell walls in all three species (Figures 2 and 3). 

 In the xylem cells, however, the LM15 was consistently detected in specific cell wall regions of the two large metaxylem cells (adjacent to the central metaxylem cell) and the cell wall of the central metaxylem cell in the vascular bundles in M. x *giganteus*. This pattern was observed to some extent in *M. sinensis* xylem cell walls and only rarely in *M. sacchariflorus* xylem cell walls (Figures 2 and 3). 

### Pectic HG is detected in cell wall of parenchyma intercellular spaces in all three *Miscanthus* species and abundantly in pith parenchyma cell walls in *M.* x *giganteus*

**Figure 4:**
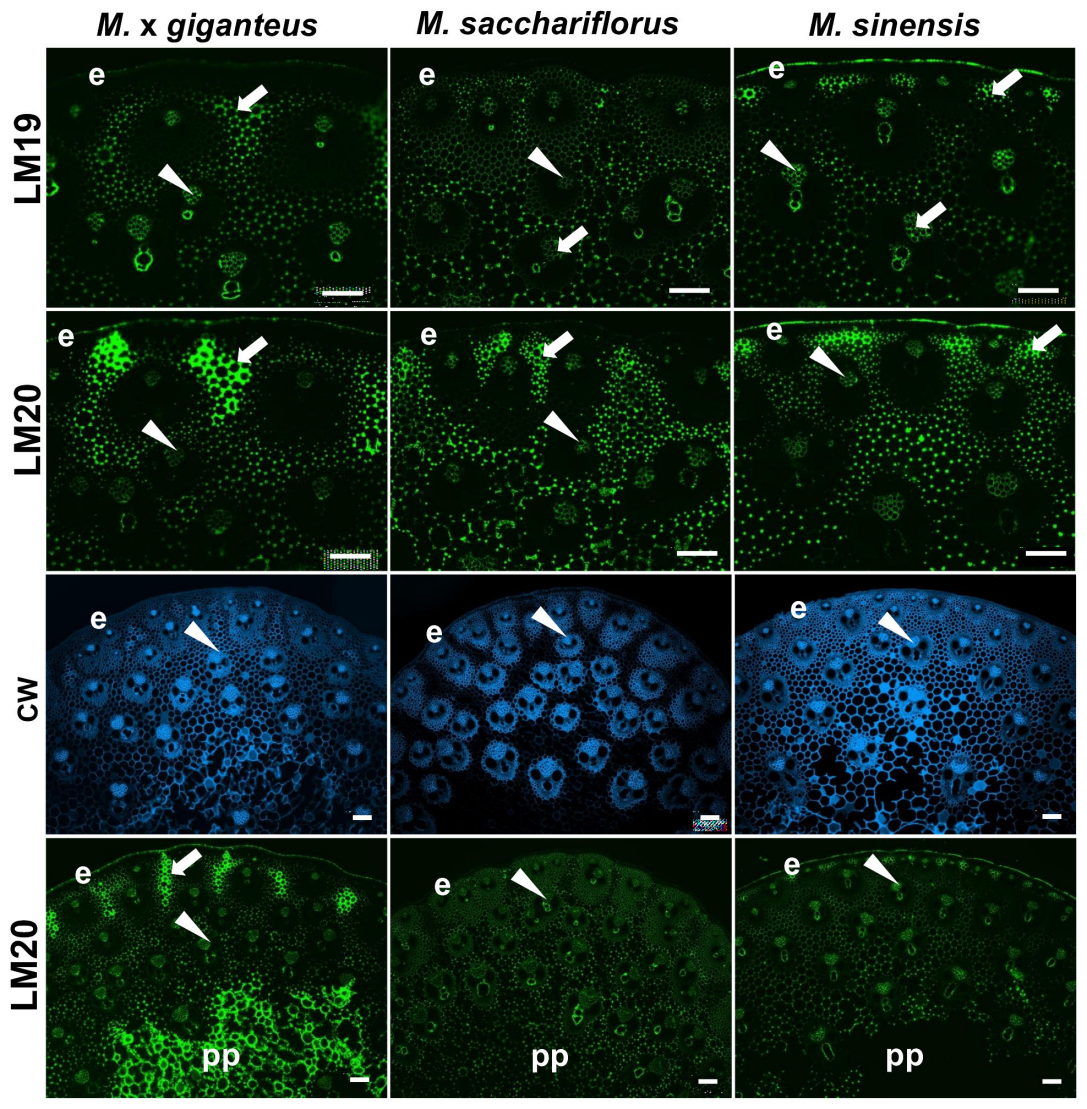
Fluorescence imaging of cell walls of equivalent transverse sections of the second internode of stems of M. **x giganteus, M**. **sacchariflorus and M. sinensis at 50 days growth**. Immunofluorescence images generated with monoclonal antibodies to pectic HG (no/low ester LM19, high ester LM20). Arrowheads indicate phloem. Arrows indicate regions of interfascicular parenchyma that are labelled strongly by the probes. Bottom six micrographs show CW staining and LM20 labelling at lower magnification to include central pith parenchyma (pp) of stems. e = epidermis. Bars = 100 µm.

The use of two monoclonal antibody probes directed to differing methyl-esterification states of pectic HG indicated that this polymer was readily detected in cell walls lining intercellular spaces in the interfascicular regions as shown for LM19 and LM20 in Figure 4. To some extent the abundance of these epitopes in these regions of parenchyma reflected the occurrence of MLG epitope abundance shown in Figure 2, as for example in the relative absence of the detection of the epitopes in the sheaths of fibre cells surrounding the vascular bundles. This correlation was particularly the case for the LM20 HG epitope in the radially extended groups of cells in M. x *giganteus* and sub-epidermal groups of cells in *M. sinensis*. In these regions the HG epitopes were detected throughout cell walls and not just in regions lining intercellular spaces. In all three species the HG epitopes were also detected in phloem cell walls and in the case of the LM19 HG epitope was detected in the cell walls of the central xylem cells. Analysis of lower magnification micrographs indicated that the LM20 high ester HG epitope was detected abundantly in all cell walls of the central pith parenchyma in M. x *giganteus* whereas this was not the case in the other two *Miscanthus* species (Figure 4). 

### Developmental dynamics of heteroxylan and MLG epitopes in *M*. x. *giganteus* stem cell walls

**Figure 5:**
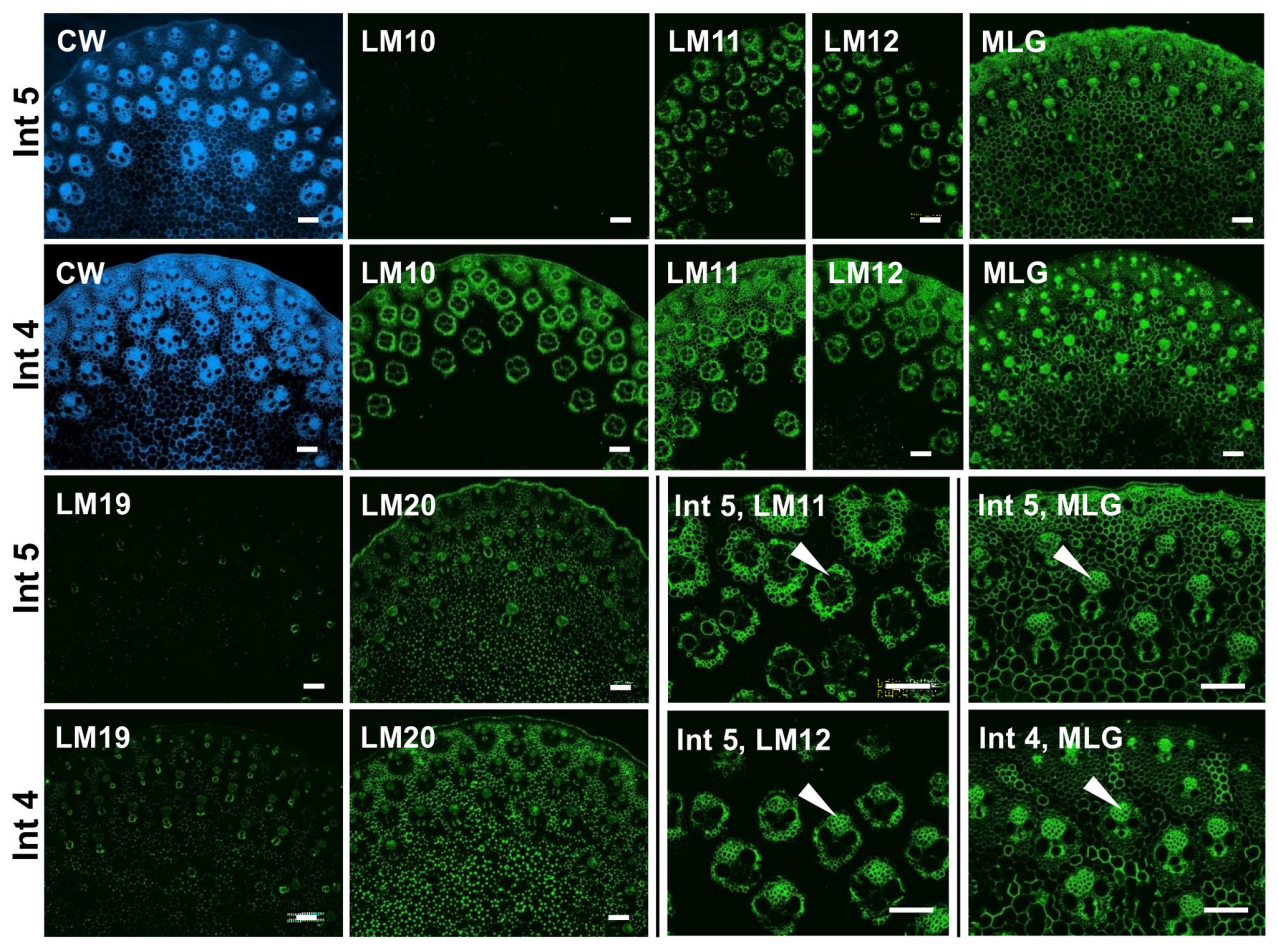
Fluorescence imaging of cell walls of equivalent transverse sections of the fourth (Int 4) and fifth (Int 5) internodes of *M*. ***xgiganteus* stems at 50 days growth**. CW staining shown in blue. Immunofluorescence images generated with monoclonal antibodies to heteroxylan (LM10, LM11 and LM12), MLG and pectic HG (no/low ester LM19, high ester LM20). Arrowheads indicate phloem. Bars = 100 µm.

The extent of the variation in detection of the heteroxylan and MLG epitopes in relation to development was explored further in M. x *giganteus* stems. Analysis of the top, middle and base of the second internode of stems at 50 days growth did not reveal any large differences in epitope occurrence. Analysis of the mid-point of more distal, younger internodes at 50 days growth indicated a decreasing gradient in the detection of the heteroxylan epitopes that was not apparent for the MLG epitope as shown in Figure 5. The LM10 xylan epitope was not detected in the youngest internode (fifth from the base) and the LM11/LM12 heteroxylan epitopes were only detected in association with the vascular bundles. At this stage the sheaths of fibre cells surrounding the vascular bundles are less developed. Relative to the LM11 epitope the LM12 epitope was detected less in the peripheral vascular bundles but detected strongly in the phloem cell walls of the more distal vascular bundles (Figure 5). In contrast, the MLG epitope was abundant in the younger internodes and particularly in the outer parenchyma regions of the youngest internode (Figure 5). In the case of the pectic HG epitopes the LM19 low ester HG epitope was less detectable in younger internodes whereas the LM20 high ester HG epitope was abundantly detected in the parenchyma cell walls (Figure 5).

### Pectic arabinan is more readily detected in *Miscanthus* stem cell walls than pectic galactan

**Figure 6:**
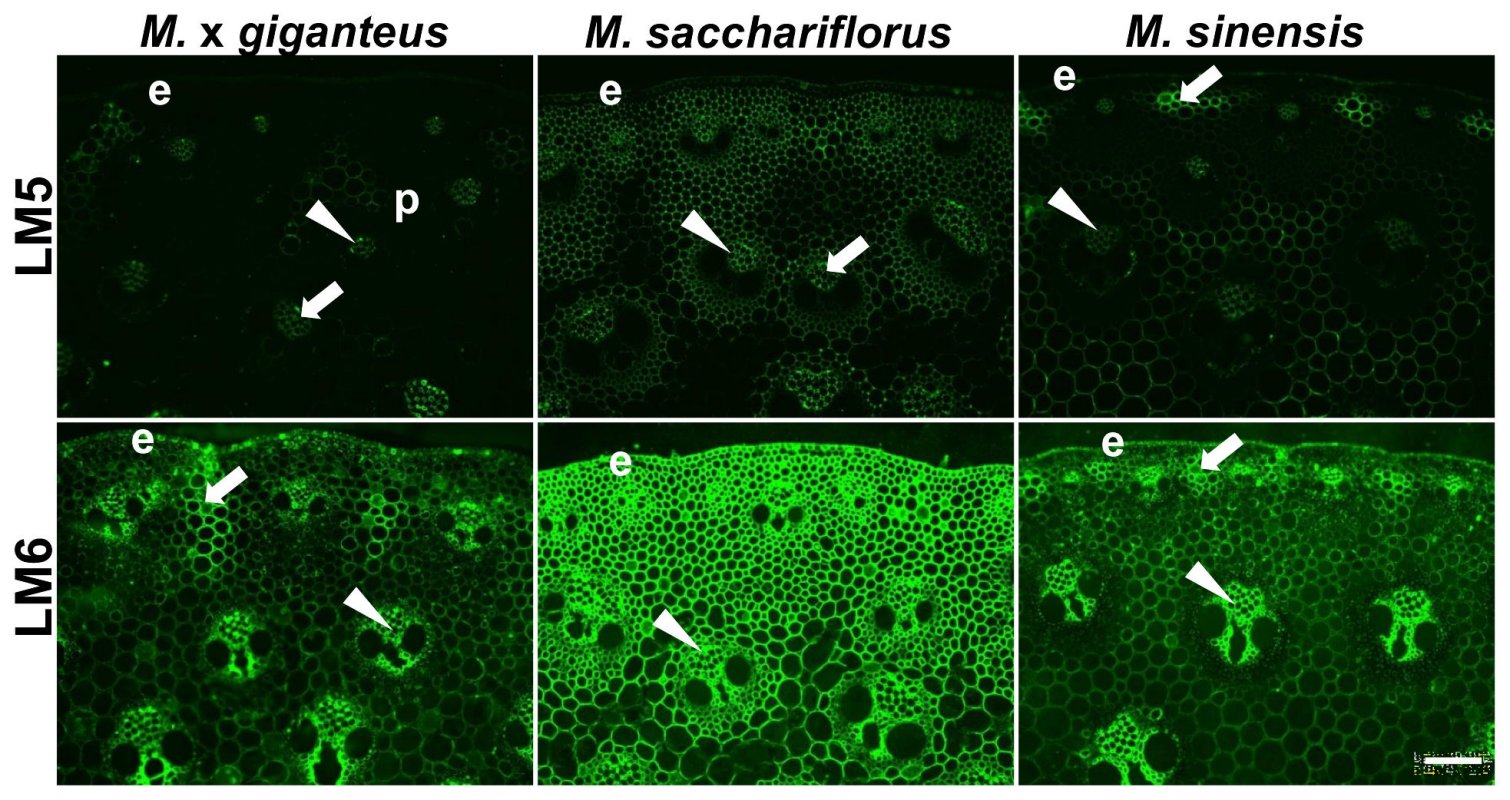
Fluorescence imaging of cell walls of equivalent transverse sections of the second internode of stems of *M*. ***xgiganteus*, *M***. ***sacchariflorus* and *M. sinensis* at 50 days growth.** Immunofluorescence images generated with monoclonal antibodies to pectic galactan (LM5) and arabinan (LM6). Arrowheads indicate phloem. Arrows indicate regions of interfascicular parenchyma that are labelled by the probes. e = epidermis. Bar = 100 µm.

*Miscanthus* stem sections obtained from the second internode after 50 days growth were analysed further for the presence of minor cell wall polysaccharide components. Analysis with probes binding to oligosaccharide motifs occurring in the side chains of the complex multi-domain pectic glycan rhamnogalacturonan-I (RG-I) revealed that the LM5 1,4-β-galactan epitope was only weakly detected in the sections and often in phloem cell walls (Figure 6). Strikingly, the LM6 1,5-α-arabinan epitope was more abundantly detected in the phloem and central vascular parenchyma cell walls and also interfascicular parenchyma regions in M. x *giganteus* and *M. sinensis* that had been identified previously by strong MLG and HG probe binding. In the case of *M. sacchariflorus* the LM6 arabinan epitope was detected abundantly and evenly in all cell walls (Figure 6). 

### Polymer masking, blocking access to specific polysaccharides, occurs in *Miscanthus* cell walls

The analyses reported above indicate a range of variations and heterogeneities in the detection of cell wall polysaccharides both across the cell types and tissue regions of an individual stem and also between equivalent stem regions of the three *Miscanthus* species that are the focus of this study. In order to explore if any of these elements of heterogeneities were related to a polysaccharide blocking probe access to other polysaccharides a series of enzymatic deconstructions were carried out prior to the immunolabelling procedures. The probes used to generate the observations reported above were applied after sections (of the second internode after 50 days growth) had been separately pre-treated with a xylanase, a lichenase (to degrade MLG), a pectate lyase (to degrade HG) or a xyloglucanase. 

**Figure 7:**
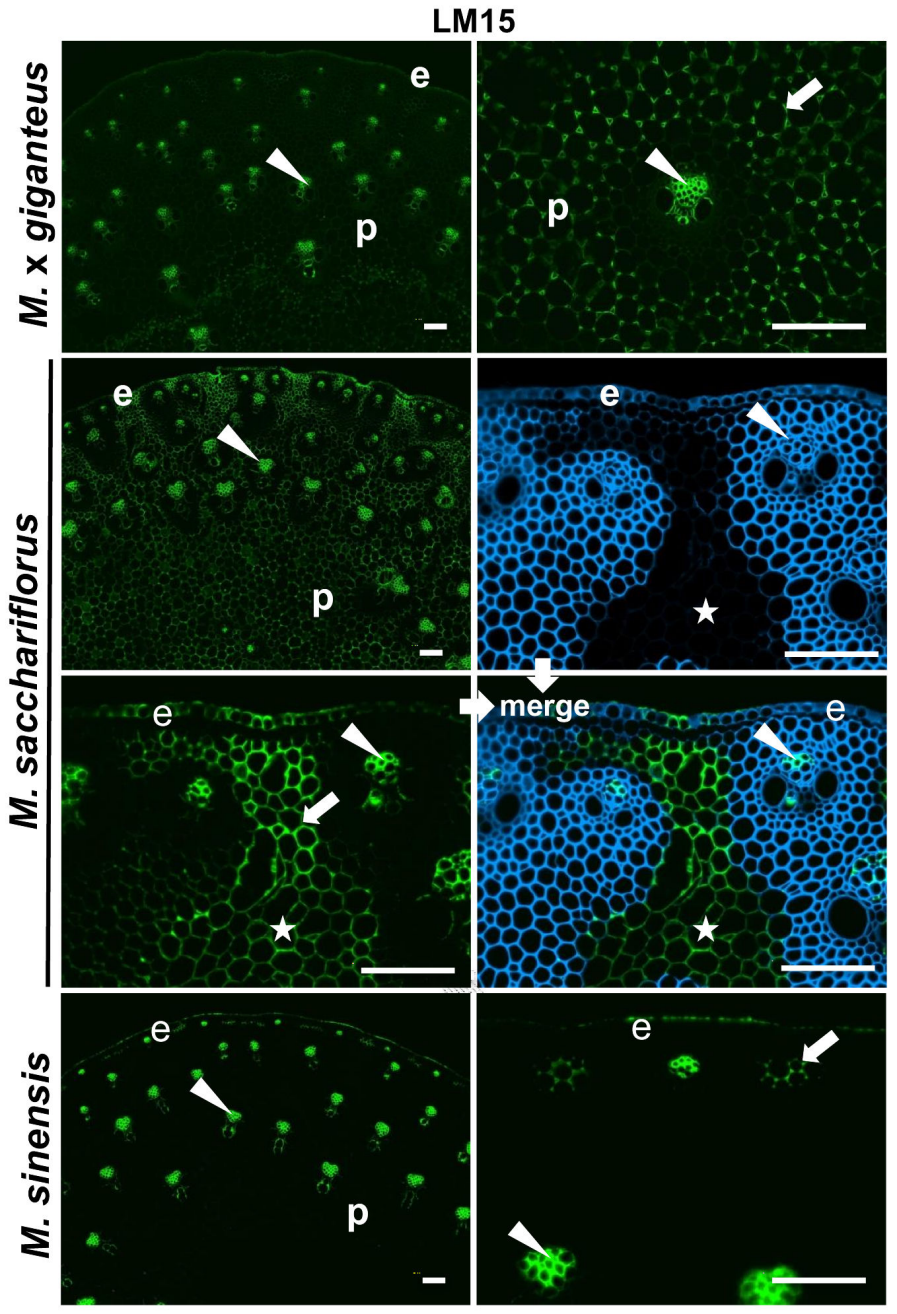
Fluorescence imaging of xylanase-treated cell walls of equivalent transverse sections from the second internode of stems of *M*. ***xgiganteus*, *M***. ***sacchariflorus* and *M. sinensis* at 50 days growth.** Immunofluorescence (FITC, green) images generated with monoclonal antibody to xyloglucan (LM15). Arrowheads indicate phloem. Arrows indicate regions of interfascicular parenchyma that are labelled by LM15. e = epidermis, p = parenchyma. Star indicates region of parenchyma in *M*. *sacchariflorus* that is unmasked and a merged image of Calcofluor White staining (blue) and LM15 labelling of the same section is shown. Bars = 100 µm.

 The only two epitopes that were notably increased in abundance and/or altered in distribution after an enzyme treatment were the LM15 xyloglucan epitope after pre-treatment with xylanase and the LM5 galactan epitope after pre-treatment with xylanase or with lichenase. Figure 7 shows low and higher magnification micrographs of LM15 binding to stem sections of all three species after enzymatic removal of xylan. In the case of xylanase-treated M. x *giganteus* cell walls the LM15 epitope was revealed to be present in cell walls lining intercellular spaces of parenchyma regions. In *M. sacchariflorus* the unmasked xyloglucan matched closely with parenchyma cell walls that did not stain with CW (Figure 7). Xylanase-unmasked LM15 epitope was less abundant in *M. sinensis* stem sections although it was observed weakly in the sub-epidermal parenchyma regions that had been identified by abundant detection of both MLG and HG and low detection of heteroxylan (Figure 7). 

**Figure 8:**
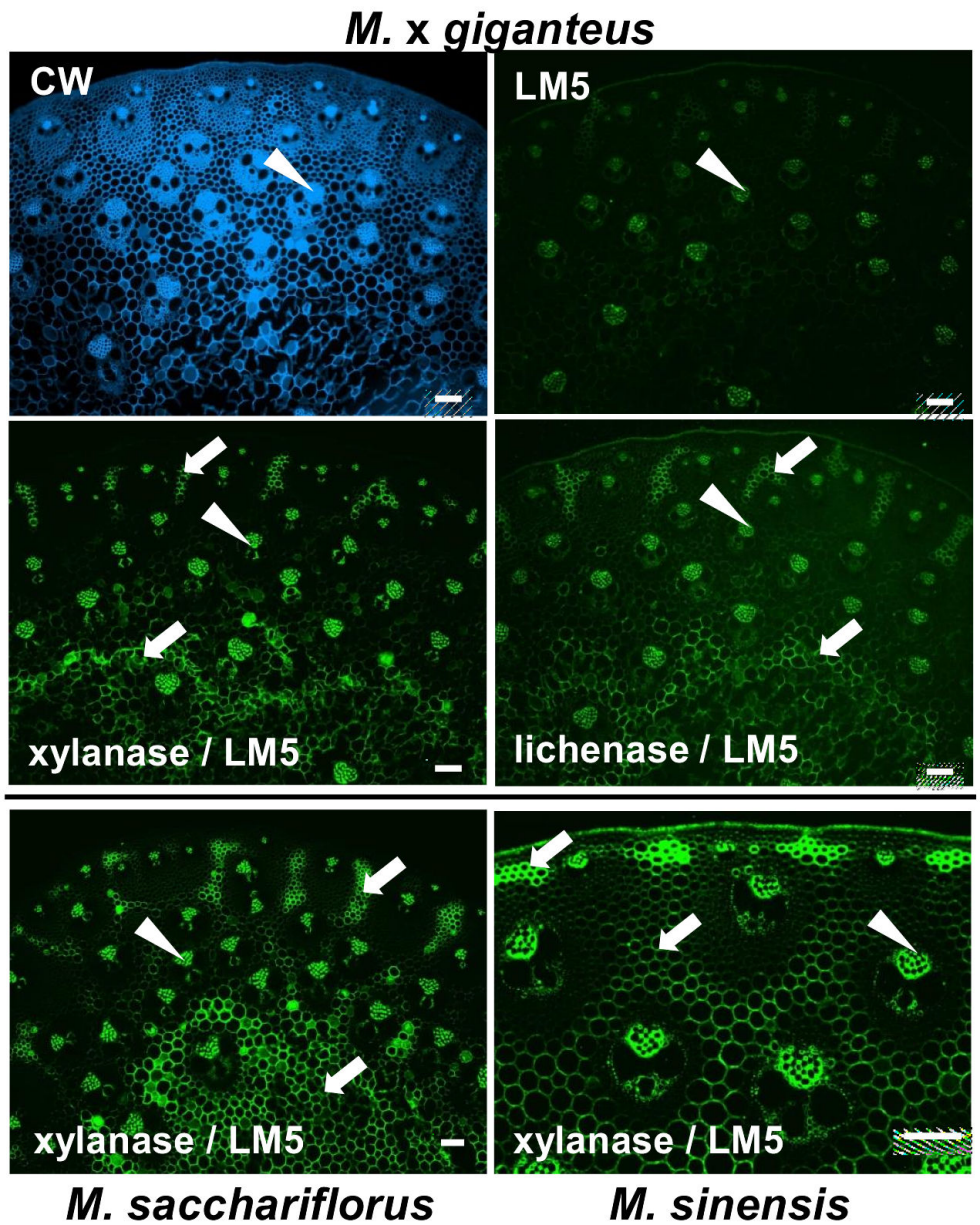
Fluorescence imaging of xylanase- and lichenase-treated cell walls of equivalent transverse sections from the second internode of stems of *M*.*x* *giganteus*, *M*. *sacchariflorus* and *M. sinensis* at 50 days growth. Immunofluorescence (FITC, green) images generated with monoclonal antibody to pectic galactan (LM5). Arrowheads indicate phloem. Arrows indicate regions of parenchyma that are labelled by LM5. Bars = 100 µm.

In the case of the LM5 galactan epitope, as shown for M. x *giganteus*, both the xylanase and the lichenase pre-treatments resulted in increased detection of the epitope in cell walls of the radially extended groups of parenchyma cells in the stem periphery, that had been identified to have a distinctive cell wall structure, and also the pith parenchyma and phloem cell walls. This increased detection of the LM5 epitope after xylanase treatment was more abundant than after lichenase treatment and this was also the case for *M. sacchariflorus* and *M. sinensis* and the patterns of LM5 epitope detection in stems of these species after xylanase treatment are shown in Figure 8. 

## Discussion

### Heterogeneity of *Miscanthus* stem cell walls

This study demonstrates that extensive cell wall molecular heterogeneity occurs in the stems of *Miscanthus* species and specifically indicates that the non-cellulosic polymers of *Miscanthus* species are not evenly detected across the cell walls of stem tissues. Mechanistic understanding of the contributions of diverse non-cellulosic polymers such as heteroxylan, xyloglucan and MLG to cell wall properties and functions in growing organs is currently limited [1,35]. Moreover, little is known of the distribution of non-cellulosic polysaccharides in the stem tissues of grasses. In maize stems *in situ* labelling studies have indicated a wide distribution of substituted xylans and with unsubstituted xylans being more restricted to secondary cell walls [36]. In rice stems, the LM10, LM11 and LM12 epitopes have restricted occurrences relating to secondary cell walls and in the same organ the MLG epitope is widely distributed [37]. It is now clear that MLG is widely present in the stems and other vegetative organs of grasses [11]. 

The major non-cellulosic glycans of *Miscanthus* stem cell walls are heteroxylans/GAXs and MLG [17,22,23]. Here, fluorescence imaging of heteroxylan and MLG, suggests a mosaic of occurrence in terms of stem anatomy with MLG being most abundantly detected in regions of low heteroxylan detection. The complementary patterns of detection of heteroxylan and MLG are observed in terms of both stem anatomy and developmental stage with MLG being most readily detected (and heteroxylan less so) in regions of interfascicular parenchyma and in younger stem tissues. MLG has been reported to increase in occurrence with the elongation of barley coleoptiles [38]. It is of interest that pectic HG epitopes are also mostly detected in the MLG-rich interfascicular parenchyma regions and in this case the epitopes are often restricted to cell wall regions lining intercellular spaces. Pectic HG is known to occur at a low level in grasses [8,15] and whether this is due to restriction to certain cell wall regions or that pectic polymers occur in other cell wall regions and cannot be detected due to low abundance, structural differences or polymer masking is not yet known. The detection of the other pectic related epitopes studied here, LM5 galactan and LM6 arabinan, which are presumed to occur within complex pectic RG-I polymers, suggest *Miscanthus* pectic molecules may be more widely distributed throughout the cell walls. It is possible, however, that the abundant widespread detection of the LM6 arabinan epitope, for example in *M. sacchariflorus*, may indicate the distribution of arabinogalactan-proteins that can also carry this epitope [39]. Considerable heterogeneity within the cell wall structures of the vascular tissues has also been detected with patterns of heteroxylan, MLG, xyloglucan and pectin epitopes all indicating varied cell wall architectures of both phloem and xylem elements. This work therefore presents the detection of cell wall heterogeneity relating to cell and tissue and organ development and indicates that cell wall biomass of *Miscanthus* is a highly heterogeneous material. How this heterogeneity changes in relation to other organs and through extended growth to harvested biomass awaits further study. The identified complementary anatomical patterning of detectable heteroxylan and MLG is also of interest in terms of the potential interactions of these glycans with cellulose microfibrils (a factor in biomass recalcitrance) as well as contributions to growth and stem properties. 

### Differences between three *Miscanthus* species

A genomic *in situ* hybridisation study suggested that M. x *giganteus* and *M. sacchariflorus* share a number of nucleotide substitutions and deletions, which could not be found in *M. sinensis* indicating that *M. sinensis* may be the most genetically distinct among the three species [40-42]. In contrast, an analysis of the cell wall composition of senesced material has indicated that M. x *giganteus* was different from the other two species [22]. The major differences between the three *Miscanthus* species used in this study in terms of cell wall stem molecular anatomies is that of the interfascicular parenchyma which is most distinctive in *M. sacchariflorus* and the high abundance of the LM20 pectic HG epitope in interfascicular and pith parenchyma of M. x *giganteus*. The interfascicular parenchyma cell walls of *M. sacchariflorus* are distinctive as they stain weakly with CW, have reduced levels of heteroxylan epitopes, particularly those of LM10 and LM12 and have relatively abundant levels of MLG and xylan-masked xyloglucan epitopes. The LM20 antibody is the most specific probe for high ester HG yet isolated [29,43] and its use indicates that the pectic HG is more methyl-esterified in the *M. giganteus* in comparison to the two parent species. Methyl-ester HG is required for cell expansion [44,45]. If this relates in any way to the faster growth rate of hybrid M. x *giganteus* is a point for future analysis. There is also the potential issue of how pectic HG can influence cell expansion in this species if it is indeed restricted to cell walls lining intercellular spaces. It is of interest in this regards that the disposition of the regions of detected unmasked xyloglucan is different in the three species – being in cell walls lining intercellular space regions in *M. giganteus* and throughout parenchyma cell walls in *M. sacchariflorus* to some extent reflecting the low heteroxylans/high MLG regions. 

### Extending the view of cell wall glycan masking

The work presented herein indicates glycan masking in cell walls of grass species. Xylanase removal of heteroxylan is effective in uncovering xyloglucan, particularly in M. x *giganteus* and *M. sacchariflorus*. It is somewhat surprising to see this effect in the regions with low/absent LM10 epitope detection - but this may indicate that only low levels of unsubstituted xylan are present in these locations and that these are effectively degraded to uncover the xyloglucan. Grass heteroxylans/GAXs are complex polymers and all potential *Miscanthus* GAX structural features, such as glucuronosyl substitutions, have not been assessed in this study due to a lack of a comprehensive set of probes. Recent work has, however, indicated that heteroxylan structure in M. x *giganteus* is comparable to that of other grasses [46]. It is of interest that xyloglucan is masked just by xylan (in regions where MLG is detected), whilst pectic 1,4-galactan is observed to be masked, in similar regions, by both xylan and MLG. The current view of glycan masking is that it is indicative of microenvironments within cell wall architectures in which a possibly non-abundant glycan can be hidden from protein/enzyme access [29]. The differential enzymatic unmasking of xyloglucan and 1,4-galactan is likely to relate to aspects of cell wall architecture and the spatial connections between these sets of polymers and is therefore suggestive of a range of differing microenvironments within a cell wall. These unmasking experiments further indicate that the parenchyma regions with abundant MLG detection have highly distinctive cell wall architectures. 

## Conclusion

The detailed *in situ* analysis of the occurrence of cell wall polysaccharides in the stems of three *Miscanthus* species has focused on the analysis of young stems, before extensive lignification, and indicates both a considerable heterogeneity across stem tissues and cell types and has also highlighted some cell wall differences between the three species. The use of cell wall degrading enzymes has extended knowledge of *Miscanthus* cell wall architectures and the potential for certain cell wall glycans to be ‘hidden’ from protein access by other glycans. This work extends understanding of *Miscanthus* cell wall diversity and properties and provides a basis to inform potential strategies for the efficient deconstruction of *Miscanthus* cell wall materials. 

## Supporting Information

File S1 **Figure S1 and S2. Figure S1.** Sampling of *Miscanthus* stem internodes. Photographs indicating sampling of stem materials from different internodes of *M*. x *giganteus*, *M. sacchariflorus* and *M. sinensis*. A: Representative stems and leaves of *Miscanthus* species at 50 days growth. B: Stems of *Miscanthus* species. C: The fourth internode (Int4) of *M*. x *giganteus* showing sampling positions of base (bm), middle (mid) and shoot (top). D: Internodes of a *M*. x *giganteus* stem. Int1 is the first internode of the stem (counting from the base), and Int6 is the youngest internode of a stem (near the shoot meristem). E and F: Internodes of stems of *M. sacchariflorus* and *M. sinensis*. Bar = 1 cm. **Figure S2.** No antibody negative control fluorescence micrographs. No-antibody negative control fluorescence micrographs showing cell walls of equivalent transverse sections of the second internode of stems of *M*. *x giganteus*, *M. sacchariflorus* and *M. sinensis* at 50 days growth. Shown for high and low magnification objectives. Images generated with Calcofluor White (CW, blue) and omission of any monoclonal antibody probe with exposure time equivalent to the longest used for antibody labelling. e = epidermis, p = interfascicular parenchyma, vb = vascular bundle, Bars = 100 µm.
(PDF)
