# Supplementary material for: Heterogeneity and Glycan Masking of Cell Wall Microstructures in the Stems of Miscanthus x giganteus, and Its Parents M. sinensis and M. sacchariflorus
Source: PLoS One. 2013 Nov 29;8(11):e82114. doi: 10.1371/journal.pone.0082114 (PMC3843723; doi:10.1371/journal.pone.0082114)

Supporting information for Xue et al. Heterogeneity and glycan masking of cell wall microstructures in the stems of *Miscanthus* x *giganteus*, and its parents *M. sinensis* and *M. sacchariflorus*

Figure S1. Sampling of *Miscanthus* stem internodes. Photographs indicating sampling of stem materials from different internodes of *M.* x *giganteus*, *M. sacchariflorus* and *M. sinensis*. A: Representative stems and leaves of *Miscanthus* species at 50 days growth. B: Stems of *Miscanthus* species. C: The fourth internode (Int4) of *M.* x *giganteus* showing sampling positions of base (bm), middle (mid) and shoot (top). D: Internodes of a *M.* x *giganteus* stem. Int1 is the first internode of the stem (counting from the base), and Int6 is the youngest internode of a stem (near the shoot meristem). E and F: Internodes of stems of *M. sacchariflorus* and *M. sinensis.* Bar = 1 cm.

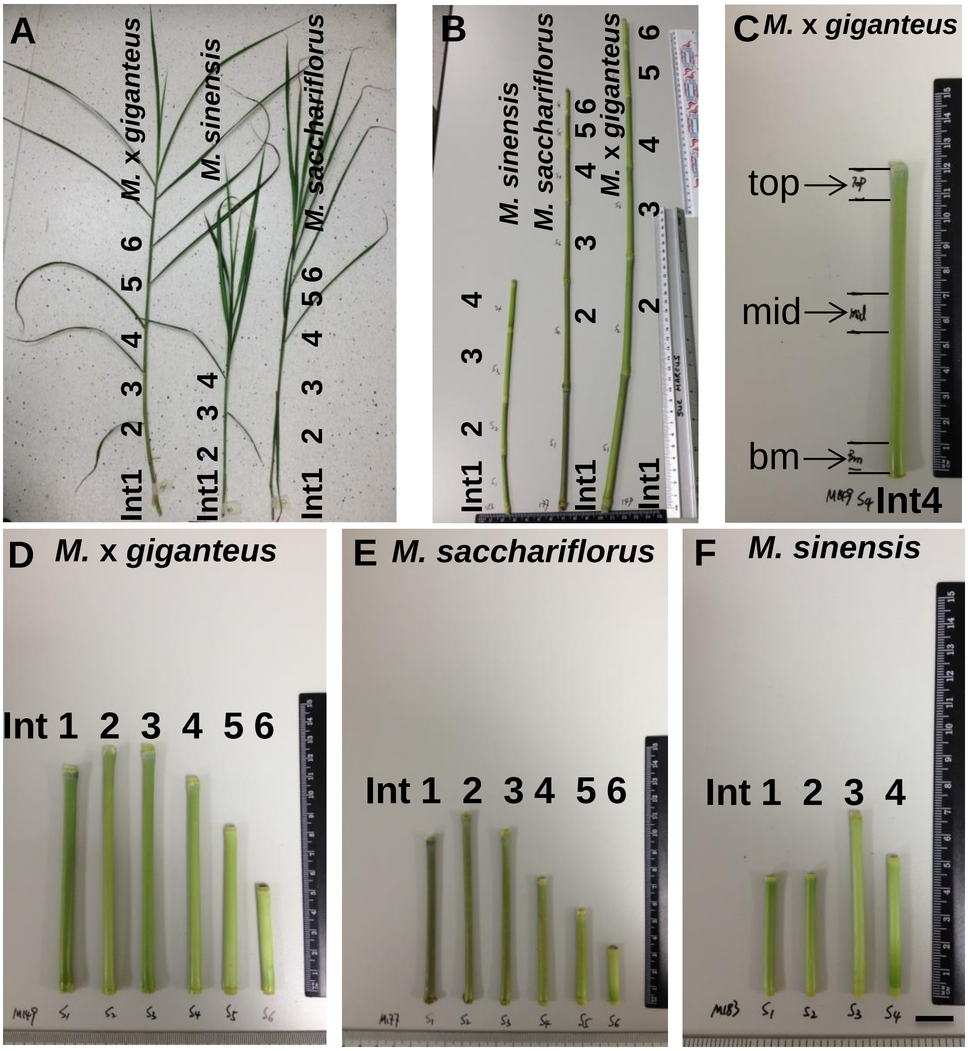

Figure S2. No antibody negative control fluorescence micrographs. No-antibody negative control fluorescence micrographs showing cell walls of equivalent transverse sections of the second internode of stems of *M. x giganteus*, *M. sacchariflorus* and *M. sinensis* at 50 days growth. Shown for high and low magnification objectives. Images generated with Calcofluor White (CW, blue) and omission of any monoclonal antibody probe with exposure time equivalent to the longest used for antibody labelling. e = epidermis, p = interfascicular parenchyma, vb = vascular bundle, Bars = 100 µm.

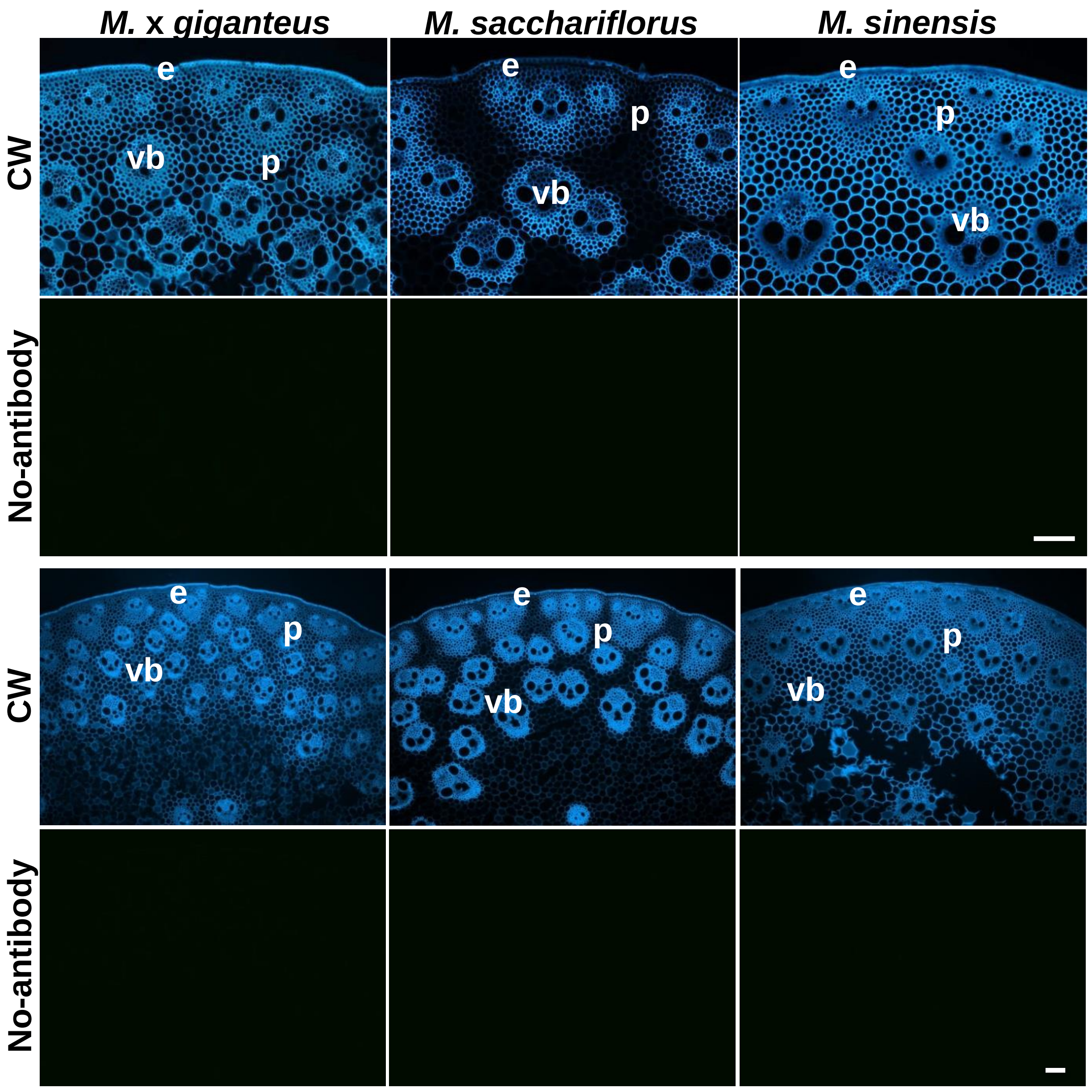

Supplement: File S1 — Figure S1 and S2. Figure S1. Sampling of Miscanthus stem internodes. Photographs indicating sampling of stem materials from different internodes of M. x giganteus, M. sacchariflorus and M. sinensis. A: Representative stems and leaves of Miscanthus species at 50 days growth. B: Stems of Miscanthus species. C: The fourth internode (Int4) of M. x giganteus showing sampling positions of base (bm), middle (mid) and shoot (top). D: Internodes of a M. x giganteus stem. Int1 is the first internode of the stem (counting from the base), and Int6 is the youngest internode of a stem (near the shoot meristem). E and F: Internodes of stems of M. sacchariflorus and M. sinensis. Bar = 1 cm. Figure S2. No antibody negative control fluorescence micrographs. No-antibody negative control fluorescence micrographs showing cell walls of equivalent transverse sections of the second internode of stems of M. x giganteus, M. sacchariflorus and M. sinensis at 50 days growth. Shown for high and low magnification objectives. Images generated with Calcofluor White (CW, blue) and omission of any monoclonal antibody probe with exposure time equivalent to the longest used for antibody labelling. e = epidermis, p = interfascicular parenchyma, vb = vascular bundle, Bars = 100 µm. (PDF) [file pone.0082114.s001.pdf]
